# Evaluation of Muscle microRNA Expression in Relation to Human Peripheral Insulin Sensitivity: A Cross-Sectional Study in Metabolically Distinct Subject Groups

**DOI:** 10.3389/fphys.2017.00711

**Published:** 2017-09-21

**Authors:** Dennis Dahlmans, Alexandre Houzelle, Johanna A. Jörgensen, Esther Phielix, Lucas Lindeboom, Matthijs K. C. Hesselink, Patrick Schrauwen, Joris Hoeks

**Affiliations:** ^1^Departments of Human Biology and Human Movement Sciences, Maastricht University Medical Center Maastricht, Netherlands; ^2^Departments of Radiology, NUTRIM School for Nutrition and Translational Research in Metabolism, Maastricht University Medical Center Maastricht, Netherlands

**Keywords:** Type 2 diabetes, insulin sensitivity, microRNA, skeletal muscle, obesity

## Abstract

In recent years, several microRNAs (miRNAs)—post-transcriptional regulators of gene expression—have been linked to the regulation of peripheral insulin sensitivity. Many of these studies, however, have been conducted in cell or animal models and the few human studies available lack adequate measurements of peripheral insulin sensitivity. In the present study, we examined the expression of 25 miRNAs, putatively involved in (peripheral) insulin sensitivity, in skeletal muscle biopsies from extensively phenotyped human individuals, widely ranging in insulin sensitivity. To identify miRNAs expressed in skeletal muscle and associated with insulin sensitivity and type 2 diabetes, a comprehensive PubMed-based literature search was performed. Subsequently, the expression of selected miRNAs was determined by RT-qPCR using predesigned 384-well Pick-&-Mix miRNA PCR Panel plates in muscle biopsies from type 2 diabetes patients, non-diabetic obese/overweight individuals, lean sedentary individuals and endurance-trained athletes. In all subjects, peripheral insulin sensitivity was measured by hyperinsulinemic-euglycemic clamp. The literature search resulted in 25 candidate miRNAs, 6 of which were differentially expressed in human type 2 diabetes compared to non-diabetic obese/overweight individuals. In turn, four of these miRNAs, i.e., miRNA27a-3p (*r* = −0.45, *p* = 0.0012), miRNA-29a-3p (*r* = −0.40, *p* = 0.0052), miRNA-29b-3p (*r* = −0.70, *p* < 0.0001) and miRNA-29c-3p (*r* = −0.50, *p* = 0.0004) demonstrated strong negative correlations with peripheral insulin sensitivity across all four subject groups. We identified miR-27a-3p and all members of the miRNA-29 family as potential regulatory players in insulin sensitivity in humans. These miRNA's may represent interesting novel targets for maintaining or improving insulin sensitivity.

## Introduction

Type 2 diabetes mellitus (T2DM) is a chronic, progressive disease and diminished insulin sensitivity in peripheral tissues is an important phenomenon in its pathogenesis. Since skeletal muscle comprises 40–60% of total body weight and accounts for 80–90% of postprandial insulin-mediated glucose uptake (DeFronzo et al., 1985; Tseng et al., 2010), muscle tissue is of high metabolic significance. Therefore, understanding the factors that may contribute to muscle insulin resistance is important in order to find new opportunities to blunt the development of insulin resistance at an early stage. In this context, recent primarily pre-clinical studies, have linked microRNAs (miRNAs) to insulin sensitivity (Mohamed et al., 2014; Bork-Jensen et al., 2015; Dahlmans et al., 2016). miRNAs are noncoding RNA molecules of 18–24 nucleotides in length that post transcriptionally regulate many cellular processes by base pairing with conventional messenger RNAs (Bartel, 2004, 2009). Furthermore, most miRNAs are predicted to target a range of messenger RNAs, and can in turn be regulated by other miRNAs, underscoring the complexity of the regulation of protein expression by miRNAs. As such, miRNAs add a post-transcriptional and pre-translational layer to the complex regulation of adaptive responses in protein content. Thus, miRNAs play an important role over a wide range of cellular functions, including the regulation of metabolism (Krutzfeldt and Stoffel, 2006).

Indeed, several miRNAs have been linked to insulin resistance in both *in vitro* and *in vivo* models for insulin resistance. For example, miRNA-135a was elevated in skeletal muscle tissue of hyperglycemic db/db mice whereas *in vivo* silencing of miRNA-135a reduced hyperglycemia and improved glucose tolerance in db/db mice, probably via inhibitory effects on IRS2 (Agarwal et al., 2013). In addition, miRNA-24 and miRNA-126 were found to be significantly upregulated in skeletal muscle of insulin resistant Goto-Kakizaki rats as compared to normoglycemic Wistar rats (Huang et al., 2009). Furthermore, miRNA-194, determined by miRNA microarray analysis, was found to be reduced in T2DM patients as well as in insulin resistant rats (Latouche et al., 2016). Knockdown of miRNA-194 in L6 muscle cells improved insulin sensitivity, an effect that coincided with improvements in mitochondrial function (Latouche et al., 2016). Along similar lines, miRNA-149, shown to positively regulate NAD+ and SIRT1 protein levels via direct interaction with poly (ADP-ribosyl) transferase-like 2 protein (PARP2), was shown to be decreased in high fat diet-induced insulin resistant mouse skeletal muscle (Mohamed et al., 2014). Finally, a study in C2C12 myotubes revealed that miRNA-106b expression was increased upon palmitic acid-induced insulin resistance while silencing of miRNA-106b improved mitochondrial health and promoted insulin sensitivity (Zhang et al., 2015). Besides these examples numerous other findings in primarily cell- and animal models link miRNAs to insulin sensitivity. In the present study, we thus performed a comprehensive literature search using the PubMed database, to select miRNAs expressed in skeletal muscle and associated with insulin resistance and T2DM.

Next, to establish human relevance of these candidate miRNAs, we aimed to examine if these miRNAs, putatively involved in insulin sensitivity, are differentially expressed skeletal muscle biopsies of thoroughly phenotyped, metabolically distinct subject groups that display a large range in peripheral insulin sensitivity and if their expression pattern could be linked to metabolic aberrations.

## Methods

### Subjects

Samples were collected from 4 previously conducted studies; all performed at the department of Human Biology and Human Movement Sciences at Maastricht University (Phielix et al., 2010, 2012; van de Weijer et al., 2014; Vosselman et al., 2015). The institutional medical ethics committee approved the aforementioned studies [clinical trial reg. no. NCT00943059 (van de Weijer et al., 2014), NCT01298375 (Vosselman et al., 2015) and NTR2002 (Phielix et al., 2010, 2012)], and all participants gave their informed written consent in accordance with the Declaration of Helsinki. Four subject groups were included in the present study: (1) overweight/obese T2DM patients (*n* = 12); (2) non-diabetic obese/overweight individuals (*n* = 12); (3) young, lean sedentary individuals (*n* = 12) and (4) young, endurance-trained athletes (*n* = 12). Subjects were selected on the availability of muscle tissue and the presence of data for peripheral insulin sensitivity and *in vivo* mitochondrial function (i.e., PCr recovery rate and VO_2_max) as main criteria. Subsequently, subjects were selected in order for the groups to match for age and BMI (i.e., lean sedentary subjects vs. endurance-trained athletes and overweight/obese, non-diabetic subjects vs. type 2 diabetic patients). All subjects were male, non-smoking and weight stable for at least 6 months. All T2DM patients were diagnosed with T2DM for at least 1 year, used metformin alone or in combination with sulfonylureas and were allowed to use statins as lipid-lowering drugs. Patients were instructed to discontinue glucose lowering medication at least the week before the actual tests and biopsies, all other medications were continued during the studies. Finally, type 2 diabetic patients were allowed to use antihypertensive medication, such as ACE inhibitors and AT-II antagonists. The non-diabetic overweight/obese individuals, did not use any medication, and were excluded if they suffered from uncontrolled hypertension, cardiovascular disease, liver dysfunction or if they had first degree relatives with T2DM. Fasting blood glucose along with oral glucose tolerance tests were performed to ensure the absence of non-diagnosed T2DM in the obese individuals. Lean, sedentary subjects were included if they participated in no more than 1 h of exercise per week for the last 2 years and if their VO_2_max was lower than 45 ml/min/kg. Endurance-trained athletes were included if they participated in endurance training at least 3 times per week for the last 2 years and if their VO_2_max was above 55 ml/min/kg. Depending on the study, body composition was either determined via hydrostatic weighing according to the method of Siri (1956), or via Dual-energy X-ray absorptiometry (DXA).

### Peripheral insulin sensitivity

Insulin sensitivity was assessed using either a two-step or a one-step hyperinsulinemic-euglycemic clamp. In total, 8 T2DM patients, 6 obese/overweight individuals, 7 lean sedentary individuals and 10 endurance-trained athletes were assessed using a two-step hyperinsulinemic-euglycemic clamp. Insulin sensitivity was assessed using a one-step clamp in 4 T2DM patients, 6 obese/overweight individuals, five lean sedentary individuals and two endurance-trained athletes.

Patients refrained from physical exercise 3 days before the glucose clamp. On the evening before the clamp, patients were provided with a standardized meal. All hyperinsulinemic-euglycemic clamps were performed after an overnight fast, the clamps were conducted according to the method of DeFronzo et al. (1979). In short, for the one-step clamp, subjects received a continuous insulin infusion of 40 mU/m^2^/min, and a parallel glucose infusion (20% Glucose) was gradually increased until a steady state in blood glucose was achieved. For the two-step clamp, participants received, a low-insulin infusion of 10 mU/m^2^/min for 3 h until a steady state in blood glucose was reached. Thereafter, a high-insulin infusion of 40 mU/m^2^/min was performed for the duration 2.5 h, after which steady state was reached. In the present study, we only present the glucose infusion rate (GIR) upon 40 mU/m^2^/min of insulin, corrected for both total body weight and lean body mass, since this is a measure for peripheral (i.e., skeletal muscle) insulin sensitivity. In our study, the expression of selected miRNAs is measured in skeletal muscle. Therefore, it is most relevant to relate the outcome of this analysis to the insulin sensitivity of that tissue.

### Mitochondrial oxidative capacity

A routine incremental bicycle test was used to assess maximal aerobic capacity (VO2max), as described previously (Kuipers et al., 1985). In addition, *in vivo* mitochondrial function in the knee extensor muscles was determined as phosphocreatine (PCrR) recovery rate, assessed by phosphorus magnetic resonance spectroscopy (31P-MRS) on a 3T whole-body MRI scanner (Achieve 3T-X, Philips Healthcare), as previously described (Lindeboom et al., 2014).

### Plasma analyses

A fasted blood sample was drawn from the antecubital vein and collected in EDTA containing tubes. After centrifugation, the plasma was frozen in liquid nitrogen, followed by long-term storage at −80°C. Plasma glucose concentrations were determined using enzymatic assays on Cobas Bio Fara and Mira analyzers (hexokinase method [Roche, Basel, Switzerland]).

### Muscle biopsies

All muscle biopsies were collected after an overnight fast and in the basal state (i.e., before any intervention). Muscle specimens were taken from the m. vastus lateralis under local anesthesia (2% lidocaine) according to the technique of Bergstrom et al. (1967) and any visible non-muscle material was dissected from muscle tissue. Subsequently, biopsies were frozen immediately in melting isopentane cooled with liquid nitrogen and stored at −80°C (Guo et al., 2001).

### miRNA selection

To identify miRNAs involved in insulin sensitivity a structured literature search was conducted between May 1st and May 24th 2016. The electronic database PubMed was consulted using the following search terms: “microRNA AND skeletal muscle AND insulin resistance OR microRNA AND skeletal muscle AND diabetes,” which resulted in 44 original research articles (reviews were excluded). Articles were only considered for further analysis if an insulin resistant or diabetic model (cell, animal or human) was used, resulting in 11 remaining articles. Subsequently, miRNAs described in those 11 studies were only included in the current study if their significantly altered expression was confirmed by either RT-qPCR or northern blotting, or when miRNAs induced significant changes on any readout for insulin sensitivity after overexpression or knockdown. Finally, miRNAs were only included when full evolutionary conservation was observed in the online database miRbase, ultimately resulting in a list of 25 miRNAs.

### miRNA isolation, cDNA synthesis and miRNA RT-qPCR

RNA extraction of the muscle biopsies was performed using the miRNeasy Mini kit (Qiagen, Germany). In a sterile glass tube, 700 μL of Trizol was combined with 10–15 mg of frozen muscle tissue and homogenized with an Ultra Turrax for 1 min at 17 000 rpm. Total RNA isolation (including small RNAs) was performed according to the manufacturer's guidelines. RNA concentrations were measured on the nanodrop ND-1000 (Biocompare, California, US) and quality was checked using a bioanalyzer (Agilent Technologies, California, US). All samples had RIN values between 7 and 10. Subsequently, RNA concentrations were diluted to a concentration of 5 ng/μl with nuclease free water. The synthetic RNA spike-in UniSp6 was added and cDNA was synthesized using the Universal cDNA Synthesis kit II (Exiqon, Denmark), according to the manufacturer's protocol. Isolated cDNA samples were analyzed by RT-PCR using predesigned 384-well Pick-&-Mix microRNA PCR Panel plates (Exiqon, Denmark), according to the manufacturer's guidelines using a CFX384 Touch Real-time PCR detection system (Bio-Rad, the Netherlands). MiRNA expression data was normalized to the geometric mean of the expression of the miR-423-3p, SNORD48 and U6snRNA housekeeper genes.

### Statistics

Subject characteristics and miRNA expression differences were assessed with the use of independent *t*-test to individually test the differences between the age- and BMI-matched subjects groups (i.e., T2DM patients vs. non-diabetic obese/overweight individuals and differences between lean sedentary individuals vs. endurance-trained athletes). Relationships between miRNA expression and peripheral insulin sensitivity were determined using Pearson correlation analysis. To test if confounding factors, such as age, BMI, fasting plasma glucose or fasting plasma insulin concentrations were contributing to the observed correlations, stepwise regression analysis was conducted using IBM SPSS Statistics for Windows (Version 24, Armonk, NY). Additionally, for each miRNA possible outliers were identified using the ROUT analysis (Motulsky and Brown, 2006), over the entire group of 48 subjects and using a cutoff value of *Q* = 1%. A *P*-value below 0.05 was considered as significantly different.

## Results

### Selecting candidate miRNAs

Through a structured literature search and selection process, we selected miRNAs previously linked to insulin resistance or T2DM. This selection process resulted in 11 publications (Table 1) and 25 miRNAs (Table 2) meeting these criteria.

**Table 1:** List of studies and miRNAs, previously demonstrated to be associated with skeletal muscle insulin sensitivity, that met the initial selection criteria of the current study.

**References**	**miRNA**	**Species**	**Validation**	**mirbase ID**
Zhou et al., 2016	Rno-miR-106b-5p	Rats	RT-qPCR	Hsa-miR-106b-5p
	Rno-miR-30d-5p			Hsa-miR-30d-5p
	Rno-miR-27a-3p			Hsa-miR-27a-3p
Latouche et al., 2016	Rno-miR-194-5p	Rats	RT-qPCR	Hsa-miR-194-5p
Bork-Jensen et al., 2015	Hsa-miR-15b-5p	Human	Micro array	Hsa-miR-15b-5p
	Hsa-miR-16-5p			Hsa-miR-16-5p
Lee et al., 2016	Rno-miR-1-3p	Rats	RT-qPCR	Hsa-miR-1-3p
	Rno-miR-16-5p			Hsa-miR-16-5p
	Rno-miR-133b-3p			Hsa-miR-133b-3p
Mohamed et al., 2014	Mmu-miR-149-5p	Mice	RT-qPCR	Hsa-miR-149-5p
	Mmu-miR-21a-5p			Hsa-miR-21-5p
Zhang et al., 2015	Mmu-miR-106b-5p	Mice	RT-qPCR	Hsa-miR-106b-5p
Agarwal et al., 2013	Mmu-miR-135a-5p	Mice	RT-qPCR	Hsa-miR-135a-5p
Chen et al., 2012	Mmu-miR-1a-3p	Mice	RT-qPCR	Hsa-miR-1-3p
	Mmu-miR-133a-5p			Hsa-miR-133a-3p
	Mmu-miR-206-5p			Hsa-miR-206
	Mmu-miR-23b-3p Mmu-miR-143-3p Mmu-miR-125a-3p			Hsa-miR-23b-3p Hsa-miR-143-3p Hsa-miR-125a-3p
Karolina et al., 2011	Rno-miR-144-3p	Rats	RT-qPCR	Hsa-miR-144-3p
	Rno-miR-146a-5p			Hsa-miR-146a-5p
	Rno-miR-150-5p			Hsa-miR-150-5p
	Rno-miR-29a-3p			Hsa-miR-29a-3p
	Rno-miR-192-5p			Hsa-miR-192-5p
Huang et al., 2009	Rno-miR-24-3p	Rats	Northern blot	Hsa-miR-24-3p
	Rno-miR-126a-3p			Hsa-miR-126-3p
He et al., 2007	Rno-miR-29a-3p	Rats	Northern blot	Hsa-miR-29a-3p
	Rno-miR-29b-3p			Hsa-miR-29b-3p
	Rno-miR-29c-3p			Hsa-miR-29c-3p

**Table 2:** Final list of all miRNA candidates included in the present study, based on a comprehensive PubMed-based literature search.

**microRNAs**	**miR base IDs**	**Mature sequences (5′–3′)**
hsa-miR-1-3p	MIMAT0000416	UGGAAUGUAAAGAAGUAUGUAU
hsa-miR-106b-5p	MIMAT0000680	UAAAGUGCUGACAGUGCAGAU
hsa-miR125a-3p	MIMAT0004602	ACAGGUGAGGUUCUUGGGAGCC
hsa-mir-126-3p	MIMAT0000445	UCGUACCGUGAGUAAUAAUGCG
hsa-miR-133a-3p	MIMAT0000427	UUUGGUCCCCUUCAACCAGCUG
hsa-miR-133b-3p	MIMAT0000770	UUUGGUCCCCUUCAACCAGCUA
hsa-miR-135a-5p	MIMAT0000428	UAUGGCUUUUUAUUCCUAUGUGA
hsa-miR-143-3p	MIMAT0000435	UGAGAUGAAGCACUGUAGCUC
hsa-miR-144-3p	MIMAT0000436	UACAGUAUAGAUGAUGUACU
hsa-miR-146a-5p	MIMAT0000449	UGAGAACUGAAUUCCAUGGGUU
hsa-miR-149-5p	MIMAT0000450	UCUGGCUCCGUGUCUUCACUCCC
hsa-miR-150-5p	MIMAT0000451	UCUCCCAACCCUUGUACCAGUG
hsa-miR-15b-5p	MIMAT0000417	UAGCAGCACAUCAUGGUUUACA
hsa-miR-16-5p	MIMAT0000069	UAGCAGCACGUAAAUAUUGGCG
hsa-miR-192-5p	MIMAT0000222	CUGACCUAUGAAUUGACAGCC
hsa-miR-194-5p	MIMAT0000460	UGUAACAGCAACUCCAUGUGGA
hsa-miR-206	MIMAT0000462	UGGAAUGUAAGGAAGUGUGUGG
hsa-miR-21-5p	MIMAT0000076	UAGCUUAUCAGACUGAUGUUGA
hsa-miR-23b-3p	MIMAT0000418	AUCACAUUGCCAGGGAUUACC
hsa-miR-24-3p	MIMAT0000080	UGGCUCAGUUCAGCAGGAACAG
hsa-miR-27a-3p	MIMAT0000084	UUCACAGUGGCUAAGUUCCGC
hsa-miR-29a-3p	MIMAT0000086	UAGCACCAUCUGAAAUCGGUUA
hsa-miR-29b-3p	MIMAT0000100	UAGCACCAUUUGAAAUCAGUGUU
hsa-miR-29c-3p	MIMAT0000681	UAGCACCAUUUGAAAUCGGUUA
hsa-miR-30d-5p	MIMAT0000245	UGUAAACAUCCCCGACUGGAAG

### Basic subject characteristics

The T2DM patients and the non-diabetic obese/overweight individuals as well as the lean sedentary individuals and the endurance-trained athletes were matched for age and BMI (Table 3). By design, the T2DM patients displayed increased fasting glucose levels (7.9 ± 0.5 mmol/L) compared to non-diabetic obese/overweight individuals (5.6 ± 0.12 mmol/L; *p* < 0.0001). Furthermore, fasting plasma glucose concentrations in sedentary individuals (5.3 ± 0.09 mmol/L) and endurance-trained athletes (5.1 ± 0.08 mmol/L) were similar (Table 3). Fasting plasma insulin was similar between the T2DM patients and non-diabetic obese/overweight individuals (20.5 ± 2.5 vs. 21.7 ± 3.8 μU/mL) as well as between the lean sedentary individuals and endurance-trained athletes (9.6 ± 1.4 vs. 7.0 ± 0.8 μU/mL) (Table 3). Body fat percentage of T2DM patients and non-diabetic obese/overweight individuals was also similar (34.7 ± 1.6 vs. 34.7 ± 2.0%) but was lower in endurance-trained athletes compared to lean sedentary individuals (12.8 ± 1.1 vs. 18.3 ± 1.1%; *p* < 0.0001; Table 3).

**Table 3:** Subject characteristics.

	**T2DM**	**Obese**	***p*-value**	**Lean**	**Athletes**	***p*-value**
Subjects	12	12		12	12	
Age (yrs)	58.83 ± 1.13	56.67 ± 2.09	0.45	22.25 ± 0.74	25.08 ± 1.25	0.05
Height (m)	1.76 ± 2.25	1.74 ± 2.16	0.24	1.83 ± 1.60	1.83 ± 2.15	0.90
Weight (kg)	101 ± 3.68	94.1 ± 3.99	0.22	73.4 ± 1.98	70.33 ± 2.14	0.31
BMI (kg/m^2^)	32.44 ± 1.02	31.83 ± 1.06	0.34	22.02 ± 0.55	20.98 ± 0.44	0.20
Body fat (%)	34.69 ± 1.63	34.65 ± 2.01	0.97	18.34 ± 1.05	12.76 ± 0.60	< 0.0001
Lean body mass (kg)	65.79 ± 1.87	60.95 ± 2.59	0.09	57.77 ± 1.78	58.98 ± 1.64	0.62
FPG (mmol/L)	7.92 ± 0.48	5.64 ± 0.12	0.0001	5.25 ± 0.09	5.13 ± 0.08	0.30
FPI (μU/mL)	20.53 ± 2.51	21.74 ± 3.83	0.79	9.59 ± 1.36	6.98 ± 0.75	0.10
HbA1c (%)	7.18 ± 0.22	5.71 ± 0.10	<0.0001	-	-	-
Triacylglycerol (mmol/L)	2.30 ± 0.32	1.31 ± 0.09	0.02	-	-	-
HDL (mmol/L)	1.03 ± 0.09	1.60 ± 0.40	0.18	-	-	-
LDL (mmol/L)	2.69 ± 0.12	3.12 ± 0.31	0.28	-	-	-
FFA (mmol/L)	0.58 ± 0.05	0.69 ± 0.14	0.49	-	-	-
PCr Recovery (s)	26.54 ± 1.69	21.26 ± 1.28	0.03	20.22 ± 1.44	15.58 ± 1.56	0.07
VO_2_max (ml/min/kg)	24.51 ± 1.13	27.81 ± 1.25	0.08	41.36 ± 0.57	59.97 ± 1.20	< 0.0001

*Age, body mass index (BMI), body fat percentage, fasting plasma glucose (FPG), fasting plasma insulin (FPI) concentrations, HbA1c, Triacyglycerol, HDL, LDL, Free-fatty acid concentrations (FFA), Phosphocreatine (PCr) Recovery and maximal aerobic capacity (${\text{VO}}_{max}^{2}$)in type 2 diabetic patients (T2DM) vs. non-diabetic overweight/obese individuals and lean sedentary individuals vs. endurance-trained athletes. Values presented are mean ± SEM*.

### Peripheral insulin sensitivity

**Figure 1:**
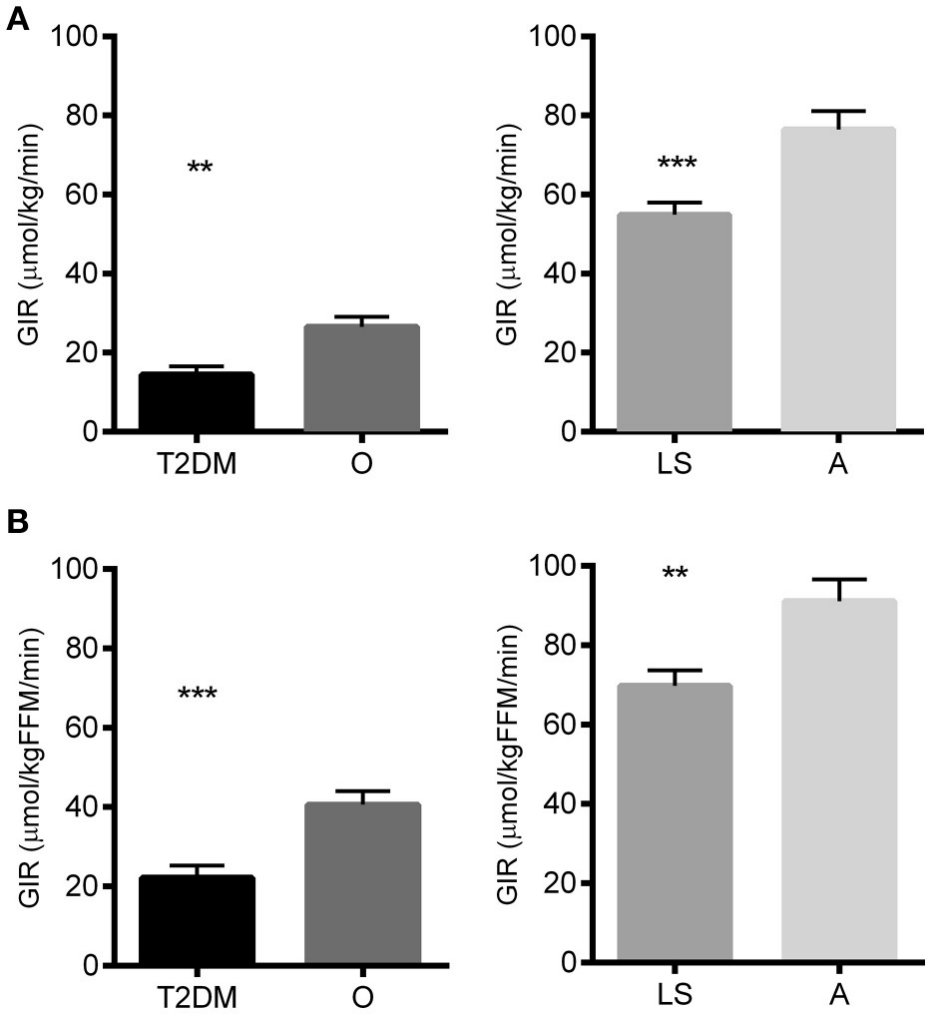
Glucose infusion rate (GIR) during the glucose clamp as a measure for peripheral insulin sensitivity in type 2 diabetes patients (T2DM), non-diabetic overweight/obese individuals (O), lean sedentary individuals (LS) and endurance-trained athletes **(A)** Glucose Infusion rate normalized to total body weight. **(B)** Glucose infusion rate normalized to the lean body mass. Significance is indicated with ^**^ and ^***^ representing *p* < 0.01 and *p* < 0.001 respectively. Graphs represent mean ± SEM.

As anticipated, peripheral insulin sensitivity, i.e., the glucose infusion rate, normalized to body weight, required to maintain euglycemia during the hyperinsulinemic-euglycemic clamp, was significantly lower in T2DM patients compared to non-diabetic obese/overweight individuals (14.5 ± 2.0 vs. 26.6 ± 2.5 μmol/min/kg, respectively; *p* = 0.0011). In turn, insulin sensitivity was significantly higher in endurance-trained athletes as compared to lean sedentary individuals (76.5 ± 4.6 vs. 54.9 ± 3.1 μmol/min/kg, respectively; *p* < 0.0001; Figure 1A). Similar results were obtained when GIR was normalized to lean body mass (Figure 1B).

### miRNAs differentially expressed between type 2 diabetic patients and non-diabetic obese/overweight individuals

**Figure 2:**
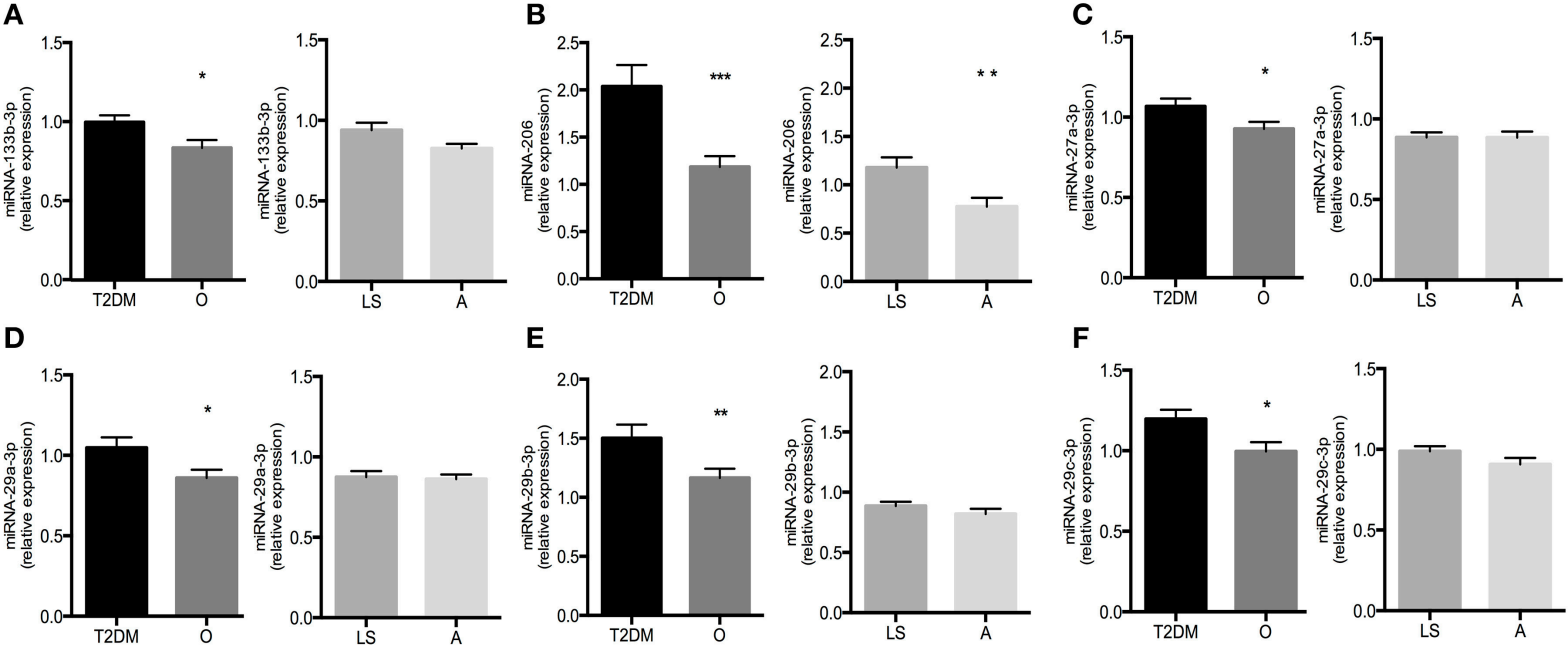
Relative expression levels of miR-133b-3p **(A)**, miR-206-5p **(B)**, miR-27a-3p **(C)**, miR-29a-3p **(D)**, miR-29b-3p **(E)**, and miR-29c-3p **(F)** in type 2 diabetic subjects (T2DM) vs. non-diabetic overweight/obese individuals (O) and lean sedentary individuals (LS) vs. endurance-trained athletes (A). Significance is indicated with ^*^, ^**^, and ^***^ representing *p* < 0.05, *p* < 0.01, and *p* < 0.001, respectively. Graphs represent mean ± SEM.

To determine which miRNAs were differentially expressed during T2DM, we compared their relative expression levels observed in T2DM patients with age- and BMI-matched non-diabetic obese/overweight individuals (Table 4). Interestingly, out of 25 miRNAs, selected based on previously reported links with insulin sensitivity, only 6 miRNAs were differentially expressed in T2DM, all of which were upregulated in T2DM patients compared to non-diabetic obese/overweight individuals (Figure 2). Thus, miRNA-133b-3p (1.00 ± 0.04 vs. 0.83 ± 0.05; *p* = 0.02), miRNA-206 (2.04 ± 0.23 vs. 1.18 ± 0.11; *p* = 0.001), and miRNA-27a-3p (1.07 ± 0.05 vs. 0.93 ± 0.04; *p* = 0.04) were all significantly higher in T2DM patients vs. non-diabetic obese/overweight individuals, respectively. Thus, the expression of miRNA-29a-3p (1.05 ± 0.07 vs. 0.86 ± 0.05; *p* = 0.03), miRNA-29b-3p (1.50 ± 0.12 vs. 1.16 ± 0.08; *p* = 0.02), and miRNA-29c-3p (1.20 ± 0.06 vs. 1.00 ± 0.06; *p* = 0.02) were also higher in T2DM patients vs. non-diabetic obese/overweight individuals, respectively (Figure 2).

**Table 4:** List of relative miRNA expressions in type 2 diabetic patients (T2DM) vs. non-diabetic overweight/obese individuals and lean sedentary individuals vs. endurance-trained athletes.

**miRNA**	**T2DM**	**Obese**	***p*-value**	**Lean sedentary**	**Athletes**	***p*-value**
hsa-miR-1-3p	0.93 ± 0.07	0.85 ± 0.05	0.34	1.05 ± 0.06	0.87 ± 0.03	0.05
hsa-miR-106b-5p	1.56 ± 0.32	1.42 ± 0.21	0.85	1.00 ± 0.10	0.80 ± 0.08	0.16
hsa-miR-125a-3p	0.97 ± 0.15	0.68 ± 0.09	0.11	0.78 ± 0.10	1.15 ± 0.15	0.06
hsa-miR-126-3p	0.83 ± 0.06	0.71 ± 0.04	0.12	0.88 ± 0.03	1.16 ± 0.06	0.001
hsa-miR-133a-3p	0.96 ± 0.07	0.84 ± 0.04	0.24	0.94 ± 0.05	0.81 ± 0.03	0.03
hsa-miR-133b-3p	1.00 ± 0.04	0.83 ± 0.05	0.02	0.94 ± 0.05	0.83 ± 0.03	0.05
hsa-miR-135a-5p	0.52 ± 0.09	0.44 ± 0.08	0.88	0.44 ± 0.10	0.69 ± 0.14	0.16
hsa-miR-143-3p	1.21 ± 0.36	0.83 ± 0.08	0.44	0.86 ± 0.09	0.94 ± 0.06	0.48
hsa-miR-144-3p	1.14 ± 0.31	1.11 ± 0.23	0.57	0.77 ± 0.14	0.44 ± 0.09	0.06
hsa-miR-146a-5p	1.78 ± 0.35	1.45 ± 0.15	0.98	1.17 ± 0.14	1.02 ± 0.17	0.08
hsa-miR-149-5p	1.43 ± 0.09	1.50 ± 0.11	0.66	1.34 ± 0.09	1.28 ± 0.13	0.28
hsa-miR-150-5p	0.85 ± 0.08	0.71 ± 0.04	0.16	0.90 ± 0.05	1.14 ± 0.07	0.01
hsa-miR-15b-5p	1.36 ± 0.29	1.33 ± 0.24	0.51	0.94 ± 0.13	0.67 ± 0.09	0.10
hsa-miR-16-5p	1.30 ± 0.27	1.17 ± 0.19	0.38	0.80 ± 0.10	0.52 ± 0.06	0.05
hsa-miR-192-5p	1.48 ± 0.28	1.38 ± 0.16	0.77	1.09 ± 0.11	1.09 ± 0.11	0.53
hsa-miR-194-5p	1.62 ± 0.38	1.46 ± 0.25	0.91	1.07 ± 0.12	1.01 ± 0.12	0.72
hsa-miR-206	2.04 ± 0.23	1.18 ± 0.12	0.003	1.18 ± 0.11	0.77 ± 0.09	0.009
hsa-miR-21-5p	1.66 ± 0.36	1.06 ± 0.06	0.23	1.03 ± 0.06	1.01 ± 0.07	0.82
hsa-miR-23b-3p	1.04 ± 0.05	0.92 ± 0.04	0.10	0.86 ± 0.03	0.98 ± 0.03	0.03
hsa-miR-24-3p	1.11 ± 0.06	0.99 ± 0.05	0.11	0.97 ± 0.04	1.03 ± 0.05	0.43
hsa-miR-27a-3p	1.07 ± 0.05	0.93 ± 0.04	0.04	0.89 ± 0.03	0.88 ± 0.04	0.97
hsa-miR-29a-3p	1.05 ± 0.07	0.86 ± 0.05	0.03	0.87 ± 0.04	0.86 ± 0.03	0.77
hsa-miR-29b-3p	1.50 ± 0.12	1.16 ± 0.08	0.02	0.89 ± 0.03	0.82 ± 0.04	0.25
hsa-miR-29c-3p	1.2 ± 0.06	1.00 ± 0.06	0.02	0.99 ± 0.03	0.91 ± 0.04	0.13
hsa-miR-30d-5p	0.76 ± 0.05	0.67 ± 0.03	0.15	0.78 ± 0.04	0.79 ± 0.03	0.96

*Values presented are mean ± SEM*.

When comparing miRNA expression levels between lean sedentary individuals and endurance-trained athletes, we found miRNA126-3p (1.16 ± 0.06 vs. 0.88 ± 0.03; *p* < 0.001) and miRNA-150-5p (1.14 ± 0.07 vs. 0.90 ± 0.05; *p* = 0.01) to be upregulated in endurance-trained athletes compared to lean sedentary individuals (Table 4).

### miRNA-27a-3p and the miRNA-29 family relate to *in vivo* peripheral insulin sensitivity

**Figure 3:**
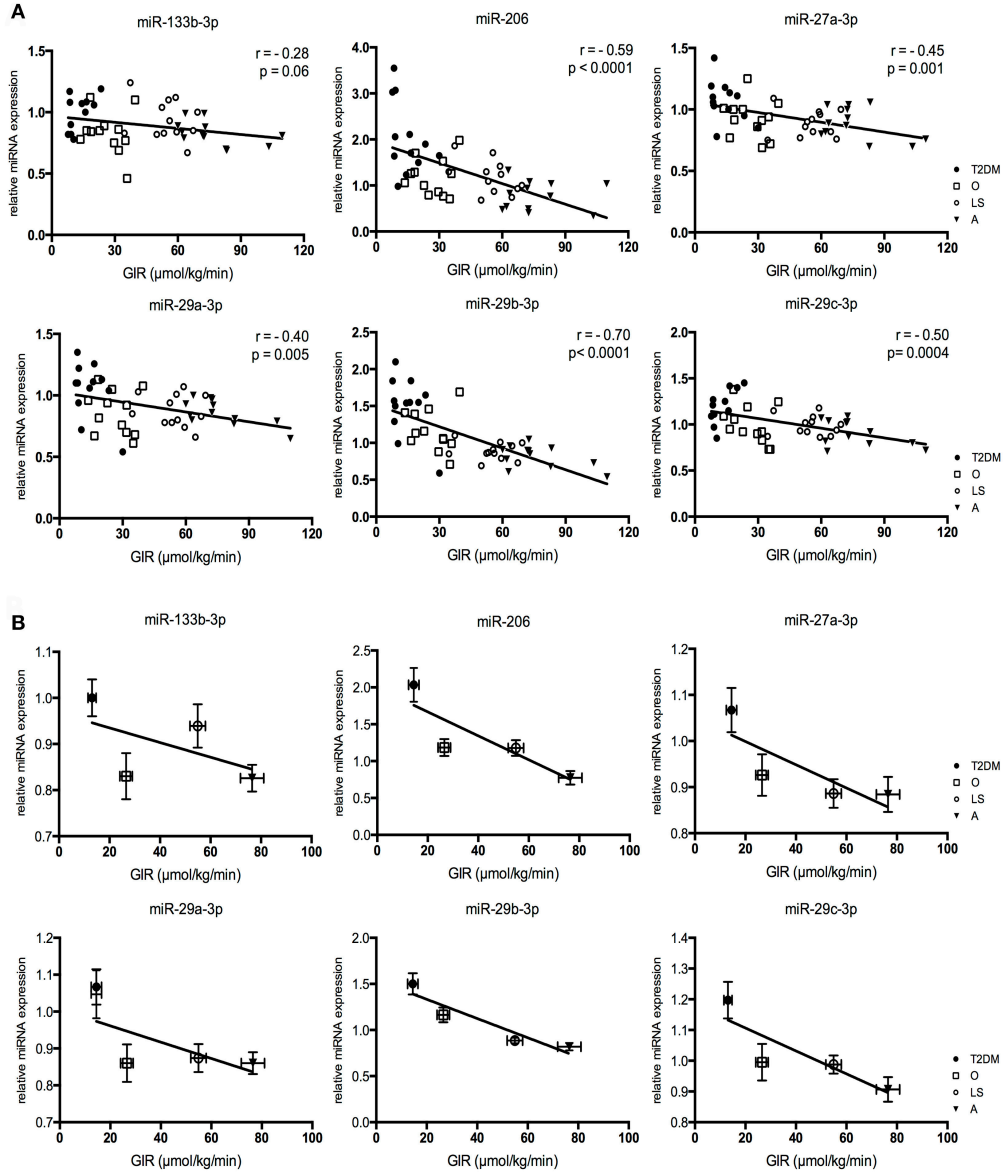
Correlations for miRNA expression levels plotted against glucose infusion rate (GIR), with the Pearson r (r) and *p*-value (p) given for each correlation **(A)**. Relative miRNA expressions vs. glucose infusion rate per subject group **(B)**. Graphs represent mean ± SEM per group.

In order to determine whether the 6 differentially expressed miRNAs in T2DM patients (relative to obese/overweight controls) also are associated with peripheral insulin sensitivity, we correlated their expression levels to the glucose infusion rate (GIR) during the hyperinsulinemic-euglycemic clamp (Figure 3A). Five miRNAs displayed a significant and negative correlation between the relative miRNA expression and the GIR during the glucose clamp (Figure 3A). The correlation between the expression of miRNA-133b-3p and GIR just did not reach significance (*r* = −0.28, *p* = 0.06). In order to visualize inter- and intra-group variation of the miRNA expressions and GIR, the correlations were also displayed per group (Figure 3B).

Our four subject groups not only differ in peripheral insulin sensitivity, but also in other subject characteristics, such as age, BMI and mitochondrial capacity (Supplemental Figure 1). To explore if the correlations of these 6 miRNAs with insulin sensitivity could also be explained by other factors we performed a stepwise linear regression analysis including VO2max, phosphocreatine recovery rate (PCrR), age, BMI, body fat percentage, fasting plasma glucose (FPG) concentrations, fasting plasma insulin (FPI) concentrations and GIR as possible determinants for miRNA expression. This analysis revealed that for miRNA-27-3p (included variable: GIR, *p* = 0.004; excluded variables: BMI, *p* = 0.23; VO2max, *p* = 0.88; PCrR, *p* = 0.45; FPG, *p* = 0.18; FPI, *p* = 0.96; age, *p* = 0.36; fat %, *p* = 0.93), miRNA-29a-3p (included variable: GIR, *p* = 0.002; excluded variables: BMI, *p* = 0.37; VO2max, *p* = 0.26; PCrR, *p* = 0.36; FPG, *p* = 0.22; FPI, *p* = 0.44; age, *p* = 1.0; fat %, *p* = 0.40), miRNA-29b-3p (included variable: GIR, *p* = 0.0001; excluded variables: BMI, *p* = 0.81; VO2max, *p* = 0.76; PCrR, *p* = 0.22; FPG, *p* = 0.10; FPI, *p* = 0.87; age, *p* = 0.07; fat %, *p* = 0.37) and miRNA-29c-3p (included variable: GIR, *p* = 0.0001; excluded variables: BMI, *p* = 0.58; VO2max, *p* = 0.36; PCrR, *p* = 0.64; FPG, *p* = 0.37; FPI, *p* = 0.56; age, *p* = 0.97; fat %, *p* = 0.51) all variables other than GIR were excluded from the model, further illustrating the relation between these miRNAs and peripheral insulin sensitivity. For miRNA-206, all variables but PCrR and FPI were excluded from the model (Included variable: PCrR, *p* = 0.001 and FPI, *p* = 0.003; excluded variables: BMI, *p* = 0.57; VO2max, *p* = 0.89; FPG, *p* = 0.18; age, *p* = 0.65; fat %, *p* = 0.84), indicating that the expression of miRNA-206 is associated with mitochondrial capacity and fasting plasma insulin concentrations.

## Discussion

MiRNAs have been reported to be involved in insulin sensitivity but information is primarily derived from preclinical cell- and animal models for insulin resistance. In the present study, we investigated 25 miRNAs, which have previously been associated with insulin sensitivity. We examined the expression of these miRNAs in subjects possessing a wide range in peripheral insulin sensitivity. We identified miRNA-27a-3p and all three members of the miRNA-29 family to be differentially regulated in muscle from patients with T2DM as compared to normoglycemic obese/overweight individuals. The expression of these miRNAs were significantly and strongly correlated to insulin sensitivity in human skeletal muscle.

Here, we show that, out of the 25 selected miRNAs, 6 candidate miRNAs (miRNA-133b-3p, miRNA-206, miRNA-27a-3p, miRNA-29a-3p, miRNA-29b-3p, and miRNA-29c-3p) were differently expressed in T2DM patients vs. non-diabetic overweight/obese individuals (Figure 2). Subsequent stepwise regression analysis of the relative miRNA expression levels and peripheral insulin sensitivity, supported the notion that these associations of miRNA-27a-3p, miRNA-29a-3p, miRNA-29b-3p and miRNA-29c-3p with peripheral insulin sensitivity were rather direct associations which were not confounded by co-correlations with other variables (i.e., VO2max, PCrR, age, BMI, body fat percentage, FPG, and FPI) that were (by design) different between these 4 phenotypically different groups (Figure 3).

The myomiR miRNA-206 was selected since it was previously reported to be downregulated in gastrocnemius muscle of high-fat diet-induced insulin resistant mice (Chen et al., 2012). In contrast to these findings, we show here that miRNA-206 expression was increased in skeletal muscle of T2DM patients as compared to non-diabetic obese/overweight individuals (Figure 2), and that miRNA-206 correlated negatively with peripheral insulin sensitivity (Figure 3). Besides obvious differences in species, it should also be noted that all subjects included in this study were weight stable for at least 6 months whereas the mice displaying differences in gene expression of miRNA-206, were in a positive energy balance and were gaining weight on a high-fat diet significantly throughout the study. Nonetheless, a possible mechanism linking miRNA-206 to insulin sensitivity is provided by a study demonstrating that reduced miRNA-206 levels increases IGF1 mRNA levels *in vivo* (Yan et al., 2013). IGF-1 has been demonstrated to stimulate insulin-like actions *in vitro*, including glucose transport, -oxidation and GLUT4 translocation (Dimitriadis et al., 1992). These findings suggest that the increased miRNA-206 expression levels we observed in T2DM patients may mediate insulin resistance by inhibiting IGF-1 expression. However, although miRNA-206 expression levels were different between T2DM patients and non-diabetic obese individuals, regression analysis revealed that the observed relationship between peripheral insulin sensitivity and miRNA-206 expression was primarily confounded by *in vivo* mitochondrial capacity and fasting plasma insulin concentrations. Interestingly, a previous study in human subjects indeed reported decreased skeletal muscle miRNA-206 expression upon 12-weeks of endurance training, which was accompanied by increases in mitochondrial capacity and insulin sensitivity (Nielsen et al., 2010), indeed pointing toward a role for miRNA-206 in the regulation of skeletal muscle mitochondrial oxidative capacity, a factor that has been repeatedly demonstrated for its involvement in insulin sensitivity (Hoeks and Schrauwen, 2012).

Previous research revealed that miRNA-27a-3p was markedly elevated in a rat model of T2DM (Zhou et al., 2016). In line with these findings, we here observed increased miRNA-27a-3p levels in skeletal muscle of T2DM patients as compared to non-diabetic overweight/obese individuals. In addition, we demonstrated a strong negative correlation between miRNA-27a-3p expression and peripheral insulin sensitivity. Additional support for the involvement of miRNA-27a-3p in the regulation of insulin sensitivity comes from *in vitro* studies in L6 muscle cells showing that miRNA-27a-3p knockdown improved insulin signaling and glucose uptake in insulin-induced insulin-resistant L6 muscle cells (Zhou et al., 2016). Furthermore, a cohort study of 163 T2DM patients and 185 healthy controls revealed that the presence of a SNP in the sequence of miRNA-27a, resulting in compromised gene expression levels, was associated with reduced risk for T2DM susceptibility (Ciccacci et al., 2013).

Finally, all members of the miRNA-29 family have previously been reported to be upregulated in several tissues, including skeletal muscle, derived from various rat models of T2DM (He et al., 2007; Karolina et al., 2011). In agreement with these findings, the present study also indicates that all three members of the miRNA-29 family (miRNA-29a-3p, miRNA-29b-3p and miRNA-29c-3p) are upregulated in skeletal muscle tissue of T2DM patients (Figure 1). Moreover, we demonstrate that all three miRNA-29 members also significantly and negatively correlated with peripheral insulin sensitivity (Figure 3), supporting recent findings in human (Massart et al., 2017). Previous research also demonstrated increased expression of miRNA-29 family members in whole blood of T2DM patients, as compared to healthy, normoglycemic controls (Karolina et al., 2011; Kong et al., 2011). Furthermore, overexpression of the miRNA-29 family in 3T3-L1 adipocytes resulted in an impaired insulin-stimulated glucose uptake and intracellular insulin signaling, whereas incubation of adipocytes with high insulin and glucose levels resulted in an upregulation of miRNA-29a and miRNA-29b (He et al., 2007). Mechanistically, the actions of the miRNA-29 family may be mediated via effects on IGF-1 (Smith et al., 2013), AKT2 (Karolina et al., 2011), Caveolin-2 (Cav2) and syntaxin-1, all molecules implicated in insulin stimulated glucose uptake in muscle cells (Oh et al., 2006) or GLUT4 vesicle-membrane interactions, respectively (Dulubova et al., 1999; Macaulay et al., 2002; He et al., 2007). Interestingly, the miR-29 family members all present a predicted binding site on at least one of the 3′UTR messenger RNAs described above.

In conclusion, we investigated 25 miRNAs for which previous, preclinical research indicated an association with insulin sensitivity, and we quantified their expression levels in skeletal muscle biopsies derived from, T2DM patients, non-diabetic overweight/obese individuals, lean sedentary individuals and endurance-trained athletes, hence representing a wide range in peripheral insulin sensitivity. We demonstrate that miRNA-27a-3p and all three members of the miRNA-29 family were upregulated in skeletal muscle of T2DM patients compared to non-diabetic overweight/obese individuals, and additionally displayed strong negative correlations with peripheral insulin sensitivity across the four metabolically distinct human subject groups. These miRNAs may therefore hold potential as novel targets in the modulation of insulin resistance.

## Author contributions

DD designed and performed the experiments, analyzed data, and wrote the manuscript. AH and JJ designed and performed the experiments and assisted in the analysis. EP, LL, and MH contributed to the initial aspects of study design and assisted in the analysis. PS and JH contributed to the design of the study, analyzed and interpreted the data and reviewed and edited the manuscript. All authors reviewed and approved the final version of the manuscript.

### Conflict of interest statement

The authors declare that the research was conducted in the absence of any commercial or financial relationships that could be construed as a potential conflict of interest.
