# Supplementary material for: Evaluation of Muscle microRNA Expression in Relation to Human Peripheral Insulin Sensitivity: A Cross-Sectional Study in Metabolically Distinct Subject Groups
Source: Front Physiol. 2017 Sep 21;8:711. doi: 10.3389/fphys.2017.00711 (PMC5613141; doi:10.3389/fphys.2017.00711)
Supplement: Supplementary file 1 [file Image1.PDF]

Supplemental figure

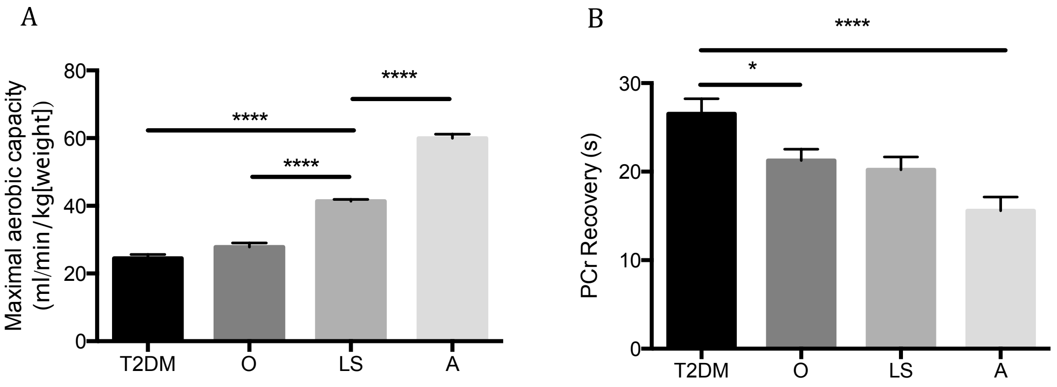

Supplemental figure 1

A) Maximal aerobic capacity (VO_2max) and **B)** phosphocreatine recovery rate (PCrR) in type 2 diabetic patients (T2DM), non-diabetic overweight/obese individuals (O), lean sedentary individuals (LS) and endurance-trained athletes (A). Significance is indicated with * and **** representing $p<0.05$ and $p<0.0001$ respectively. Values presented are mean ± SEM.
